# A Case of *Serratia marcescens* Endocarditis in a Nonintravenous Drug-Using Male Patient and Review of Literature

**DOI:** 10.1155/2019/3715404

**Published:** 2019-06-20

**Authors:** Achilleas Nikolakopoulos, Nikolaos Koutsogiannis, Panagiota Xaplanteri, Charalambos Gogos, Fevronia Kolonitsiou, Alexandra Lekkou

**Affiliations:** ^1^Department of Internal Medicine, University Hospital of Patras, 26504 Patras, Greece; ^2^Department of Cardiology, University Hospital of Patras, 26504 Patras, Greece; ^3^Department of Microbiology, University Hospital of Patras, 26504 Patras, Greece; ^4^Department of Infectious Diseases, University Hospital of Patras, 26504 Patras, Greece

## Abstract

**Introduction:**

*Serratia marcescens* is a rare cause of infective endocarditis and has almost exclusively been associated with intravenous drug use and hospital-acquired infections. Here, we present a case of infective endocarditis caused by *Serratia marcescens* in an otherwise healthy, nonintravenous drug-using male patient.

**Case Report:**

A 41-year-old man presented with hypertension and hemoptysis. Blood cultures were obtained that showed bacteremia by *Serratia marcescens*. An echocardiogram was carried out that revealed severe mitral regurgitation accompanying ruptured mitral chordae tendineae. The patient received the appropriate antibiotic treatment, without further surgical intervention.

**Discussion:**

Hospital-acquired infections by *Serratia* species are a common problem in medical practice and have been attributed to specialized interventional procedures. Taking into consideration the patient's immunocompetence and lack of intravenous drug use, it is possible that bacteremia could be attributed to a medical procedure. Moreover, in contrast to most cases described in the literature, no surgery was performed.

## 1. Introduction

*Serratia marcescens*, an aerobic (facultative anaerobe), oxidase-negative, non-lactose-fermenting Gram-negative bacillus, is a rare cause of infective endocarditis and has almost exclusively been associated with intravenous drug use and hospital-acquired infections. Here, we present a case of infective endocarditis caused by *Serratia marcescens* in an otherwise healthy, nonintravenous drug-using male patient.

## 2. Case Report

**Figure 1:**
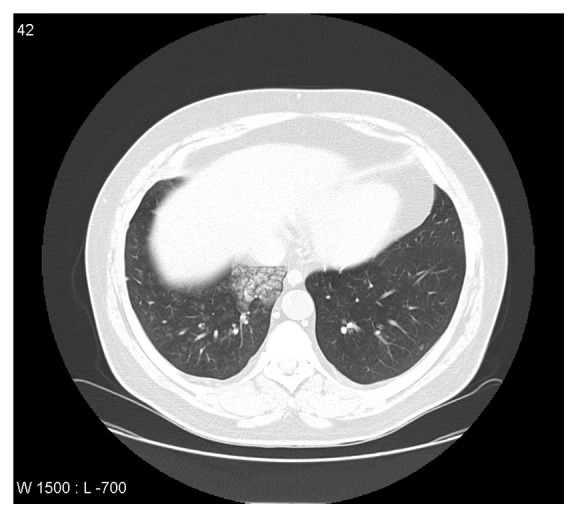
Chest CT scan showing alveoral hemorrhage and consolidation in the right middle lobe.

The patient is a 41-year-old man, without any history of disease or medication. He is slightly obese and a current smoker (>50 pack-years) and reports moderate alcohol consumption. He was admitted in a regional hospital with sudden onset of hemoptysis and headache. There, the patient was diagnosed with hypertension (systolic blood pressure: 240 mmHg, diastolic blood pressure: 110 mmHg), and hemoptysis was confirmed. A brain and chest computed tomography (CT) scan was performed that showed no findings from the brain and alveoral hemorrhage, respectively (Figure 1). In addition, the patient underwent a formal transthoracic echocardiogram (TTE), which revealed mild to moderate mitral valve regurgitation and prolapse. Blood tests were within normal rates. The patient was subsequently transported to the Pulmonary Department of our hospital for further investigation and treatment.

Upon his admission, the patient's temperature was 37.6°C, heart rate was 98 bpm, respiratory rate was 22 breaths per minute, blood pressure was 177/89 mmHg, and an arterial blood gas showed mild hypoxemia in room air (pH = 7.47; pCO_2_ = 34 mmHg; pO_2_ = 68 mmHg; HCO_3_ = 18 mmol/L; O_2_ saturation = 93%). Blood tests were normal again, except for elevated uric acid levels. Blood cultures were collected, and he was initially started on empiric broad-spectrum antibiotic treatment of piperacillin-tazobactam and oxygen supplementation due to hypoxemia. Chest CT was performed once again, which identified findings compatible with alveoral hemorrhage and consolidation in the right middle lobe. An abdominal CT scan resulted in findings such as slightly enlarged liver without focal lesions and bilateral, well-defined adrenal lesions, possibly adenomas. The patient refused to undergo a new brain CT or MRI scan. Pulmonary function tests showed obstructive lung disease, and bronchoscopy revealed blood clots in the right middle lobe. His HIV-screening test was negative, as was Mantoux tuberculin skin test (TST).

**Figure 2:**
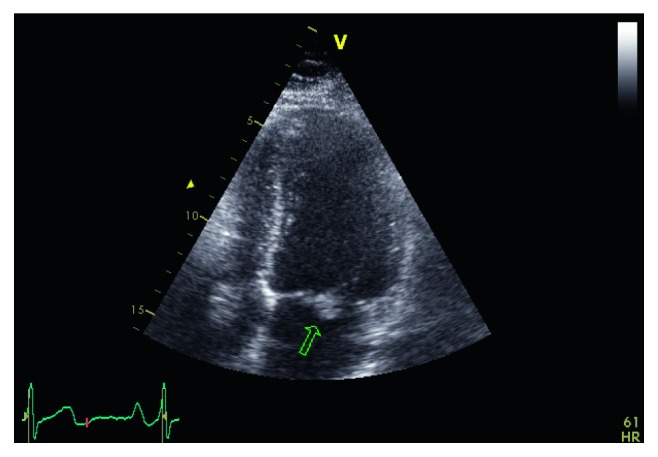
Echocardiogram showing ruptured mitral chordae tendineae.

**Figure 3:**
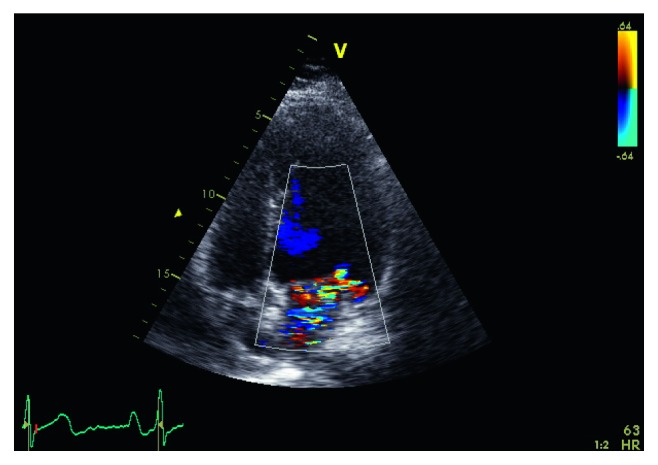
Echocardiogram showing severe mitral regurgitation.

Two blood cultures revealed bacteremia by *Serratia marcescens*, and this finding was confirmed with one more blood culture, taken 5 days later. The *Serratia marcescens* isolate was susceptible to cefepime, meropenem, gentamicin, sulfamethoxazole-trimethoprim, and ciprofloxacin. An urgent transesophageal echocardiogram (TEE) was performed which demonstrated severe mitral regurgitation accompanying ruptured mitral chordae tendineae (Figures 2 and 3). The patient was transported to the Infectious Diseases Department, and antibiotic treatment was subsequently changed to meropenem 2 g tid, ciprofloxacin 400 mg bid, and gentamicin 80 mg tid intravenously, according to antibiogram. Of notice, he reported a diagnostic arthrocentesis in his left knee joint a few weeks ago.

Three days later, the patient was afebrile, hypoxemia and hemoptysis were resolved, blood tests remained within normal limits, including inflammation markers such as C-reactive protein (CRP) and WBC, and multiple subsequent blood culture results came back negative. A new transesophageal echocardiogram was performed 20 days after the previous one, without further deterioration.

The patient, after having stayed at our hospital for 4 weeks, was transported back to the regional hospital in order to continue his intravenous antibiotic treatment with meropenem and ciprofloxacin for a total of 6 weeks. He had already completed 2 weeks of gentamicin treatment.

Both cardiologist and cardiac surgeons suggested surgery for valve repair or replacement, but the patient refused. Therefore, close surveillance with repeat echocardiograms every 3–6 months was recommended. Moreover, pulmonary surveillance with a repeat bronchoscopy was advised after completion of therapy. After 24 months, he is currently asymptomatic, without limitations in physical activity.

## 3. Discussion

Community-acquired infective endocarditis due to *Serratia* species is almost exclusively seen in intravenous drug users. Most commonly, infection implicates the aortic and mitral valves, frequently occurring on previously undamaged cardiac valves. Septic embolization is also common. Antibiotic treatment alone is usually insufficient, and surgical replacement of the involved valve is needed [1–7].

Hospital-acquired infections by *Serratia* species are a common problem in medical practice and have been attributed to specialized interventional procedures such as surgery, bronchoscopy, and foreign body placement, as well as routine hospitalization practices, through contaminated vials and other materials [8–11]. Its ability to survive and grow under extreme conditions, including in disinfectants, antiseptics, and double-distilled water, probably facilitates nosocomial infections. Contamination can occur whenever between medication and material manufacturing by pharmaceutical manufacturers or compounding pharmacies and administration to the patient. Use of single-use medication vials on multiple patients, use of a common syringe for multiple medications/patients, and use of multidose vials have all been implicated in outbreaks of nosocomial infections [12–14]. In most cases, lack of adequate sterile conditions and hand hygiene, in particular, is the common cause. Patients most at risk are those in intensive care units who are subjected to medical devices, especially central venous catheters, and treated with broad-spectrum antimicrobial drugs. Outbreaks in neonatal wards have frequently been reported, as well [15, 16]. Contamination of materials and devices, such as bronchoscopes, could be attributed to inadequate or erroneous cleaning/sterilization procedures, or even manufacturing defects [17, 18].

*Serratia marcescens* possesses a number of virulence factors, such as fimbriae or fimbria-like adhesins, which mediate surface attachment and biofilm formation and likely increase the opportunities of this organism to infect humans through attachment to abiotic surfaces [19].

Infections caused by *Serratia marcescens* may be difficult to treat because of resistance to a variety of antibiotics, including ampicillin and first-, second-, and third-generation cephalosporins. Moreover, imipenem-resistant strains exhibiting *β*-lactamase production have also been reported, that may become prevalent in the near future, further hindering treatment. Resistance to *β*-lactams has been described to be mediated through two distinct mechanisms: first, high-level production of chromosomal AmpC cephalosporinases combined with substantially decreased outer-membrane permeability, and second, synthesis of *β*-lactamases able to hydrolyse carbapenems. Aminoglycosides have good activity against *Serratia marcescens*, but resistant strains have also been reported. Alterations in the cell envelope prevent uptake of the drug, and also the drug itself can be modified by inactivating enzymes that adenylate, acetylate, or phosphorylate the hydroxyl or amino groups of aminoglycosides. This type of resistance is commonly mediated by plasmids and is often transferable [20].

Taking into consideration the patient's immunocompetence and lack of intravenous drug use, it is possible that bacteremia could be attributed to a medical procedure. One possible scenario is that the infection occurred during the arthrocentesis procedure he was subjected to, a few weeks before his hospitalization. This presumes that the first echocardiogram performed 2 weeks before his arrival, at the regional hospital, misidentified ruptured mitral chordae tendineae for mitral valve prolapse. Another scenario is that the infection occurred during his current hospitalization, where bacteremia resulted in infective endocarditis and ruptured mitral chordae tendineae of an already faulty mitral valve with prolapse. Although *Serratia* species have been associated with bronchoscopes and the procedure of bronchoscopy, in this case, bronchoscopy was preceded by the blood culture test, so it is unlikely that this could be the route of entry.

Finally, it is of notice that, in contrast to most cases described in the literature, no surgery was performed. Endocarditis by *Serratia* species has a poor prognosis, and surgical management is often required. In this case, the patient received only antibiotic treatment and survived, while remaining asymptomatic.
